# Inflammation and Aortic Pulse Wave Velocity: A Multicenter Longitudinal Study in Patients With Inflammatory Bowel Disease

**DOI:** 10.1161/JAHA.118.010942

**Published:** 2019-02-02

**Authors:** Luca Zanoli, Kadir Ozturk, Maria Cappello, Gaetano Inserra, Giulio Geraci, Antonio Tuttolomondo, Daniele Torres, Antonio Pinto, Andrea Duminuco, Gaia Riguccio, Musa B. Aykan, Giuseppe Mulé, Santina Cottone, Alessandra F. Perna, Stephane Laurent, Pasquale Fatuzzo, Pietro Castellino, Pierre Boutouyrie

**Affiliations:** ^1^ Nephrology Department of Clinical and Experimental Medicine University of Catania Italy; ^2^ Department of Gastroenterology Gulhane School of Medicine Etlik, Ankara Turkey; ^3^ DIBIMIS School of Medicine University of Palermo Italy; ^4^ Internal Medicine Department of Clinical and Experimental Medicine University of Catania Italy; ^5^ Unit of Nephrology and Hypertension Department of Internal Medicine University of Palermo Italy; ^6^ Department of Internal Medicine Gulhane School of Medicine Etlik, Ankara Turkey; ^7^ First Division of Nephrology Department of Cardiothoracic & Respiratory Sciences University of Campania “Luigi Vanvitelli” Naples Italy; ^8^ Department of Pharmacology HEGP Université Paris Descartes AP‐HP INSERM U970 Paris France

**Keywords:** arterial stiffness, Crohn disease, inflammation, pulse wave velocity, tumor necrosis factor‐alpha, ulcerative colitis, Hypertension, Vascular Disease

## Abstract

**Background:**

Inflammatory bowel disease (IBD) is characterized by a low prevalence of traditional risk factors, an increased aortic pulse‐wave velocity (aPWV), and an excess of cardiovascular events. We have previously hypothesized that the cardiovascular risk excess reported in these patients could be explained by chronic inflammation. Here, we tested the hypothesis that chronic inflammation is responsible for the increased aPWV previously reported in IBD patients and that anti‐TNFa (anti‐tumor necrosis factor‐alpha) therapy reduce aPWV in these patients.

**Methods and Results:**

This was a multicenter longitudinal study. We enrolled 334 patients: 82 patients with ulcerative colitis, 85 patients with Crohn disease, and 167 healthy control subjects matched for age, sex, and mean blood pressure, from 3 centers in Europe, and followed them for 4 years (range, 2.5–5.7 years). At baseline, IBD patients had higher aPWV than controls. IBD patients in remission and those treated with anti–TNFa during follow‐up experienced an aortic destiffening, whereas aPWV increased in those with active disease and those treated with salicylates (*P*=0.01). Disease duration (*P*=0.02) was associated with aortic stiffening as was, in patients with ulcerative colitis, high‐sensitivity C‐reactive protein during follow‐up (*P*=0.02). All these results were confirmed after adjustment for major confounders. Finally, the duration of anti–TNFa therapy was not associated with the magnitude of the reduction in aPWV at the end of follow‐up (*P*=0.85).

**Conclusions:**

Long‐term anti–TNFa therapy reduces aPWV, an established surrogate measure of cardiovascular risk, in patients with IBD. This suggests that effective control of inflammation may reduce cardiovascular risk in these patients.

**Clinical Perspective**

**What Is New?**

- A long‐term anti–tumor necrosis factor‐alpha (TNFα) therapy in patients with inflammatory bowel disease (IBD) reduces aortic pulse‐wave velocity to a level comparable to that of healthy individuals, whereas high doses of salicylates lead to aortic stiffening.

**What Are the Clinical Implications?**

- Effective control of inflammation may reduce cardiovascular risk in patients with inflammatory bowel disease as it improves aortic pulse‐wave velocity, an established surrogate measure of cardiovascular risk.

Patients with inflammatory bowel disease (IBD) have a low prevalence of traditional risk factors for cardiovascular disease^1^, ^2^, ^3^, ^4^ together with an excess of cardiovascular events.^5^ We previously proposed that at least a part of the difference between expected and observed cardiovascular risk reported in these patients could be caused by chronic severe inflammation and mediated by an increase in aortic pulse‐wave velocity (aPWV),^6^ a well‐established and independent cardiovascular risk marker^7^ and an intermediate end point for cardiovascular events.^8^ Three aggregate data meta‐analyses of cross‐sectional studies have confirmed that aPWV and reflected waves are increased in patients with IBD.^9^, ^10^, ^11^ A recent meta‐analysis of individual participant data from cross‐sectional studies has extended these results in several subgroups of patients with IBD, including those without known cardiovascular risk factors, and the study reported that aPWV was associated with disease duration, a factor associated with the burden of inflammation over time, even after adjustment for major confounders.^12^ Finally, an association between increased aortic stiffness and left ventricular dysfunction was recently reported in patients with IBD,^13^ supporting the hypothesized link between chronic inflammation, arterial stiffening, and cardiovascular events in these patients.

In a meta‐regression of 8 cross‐sectional studies^9^ and a pilot longitudinal study of 32 patients with IBD,^14^ we hypothesized that the increased arterial stiffness detected in patients with IBD could be at least partly reversed by anti–tumor necrosis factor‐alpha (anti‐TNFα) therapy, as already reported in another model of chronic inflammation.^15^

In this multicenter longitudinal prospective study, we hypothesized that chronic inflammation is responsible for the increased aPWV previously reported in patients with IBD and that anti‐TNFα drugs could reduce aPWV. Consequently, the aim of the present study was to investigate the role of inflammation and therapy on aPWV in patients with IBD.

## Methods

The data, analytic methods, and study materials will be made available from the corresponding author to other researchers for purposes of reproducing the results or replicating the procedure.

### Patient Selection

This study was a multicenter, open‐label longitudinal prospective study conducted in the Department of Clinical and Experimental Medicine of the University of Catania, Italy; the DIBIMIS of the University of Palermo, Italy; and the Department of Gastroenterology of the Gulhane School of Medicine of Ankara, Turkey.

Sequential patients with an established clinical and endoscopic diagnosis of IBD were recruited at our departments. Concomitantly, healthy volunteers matched for age, sex, and mean blood pressure (BP) (ratio of 1 control per IBD patient) were randomly selected from our local community databases and invited to take part in the study. Individuals with previous cardiovascular disease (coronary heart disease, stroke, and transient ischemic attack) and those with diabetes mellitus, chronic kidney disease, and infectious and inflammatory disorders other than Crohn disease (CD) and ulcerative colitis (UC) were excluded, as well as those on treatment for hypertension or dyslipidemia. Written informed consent was obtained from each patient included in the study. The study protocol conformed to the ethical guidelines of the 1975 Declaration of Helsinki and was previously approved by the Ethics Committee on Research on Humans of the University of Catania.

### Study Design

All participants were studied in a quiet room with a controlled temperature of 22±1°C after 15 minutes of recumbent rest. In each subject, a noninvasive hemodynamic study was performed by an expert operator blinded to the clinical data and therapy. A second operator, blinded to the hemodynamic examination, collected the clinical data using a standardized questionnaire.

### Hemodynamic Data

A hemodynamic examination was performed at baseline and at the end of follow‐up. Brachial BP measurements were performed using an oscillometric device (Dinamap ProCare 100 [GE Healthcare, Milwaukee, WI] in the centers of Catania and Palermo, Italy, and an arteriograph device [TensioMed Ltd, Budapest, Hungary] in the third center). The carotid‐femoral (aortic) pulse wave velocity was measured by a SphygmoCor device (SphygmoCor system^®^; AtCor Medical, Sydney, Australia) in two centers (Catania and Palermo, Italy) using the foot‐to‐foot velocity method, the intersecting tangent algorithm, and the direct distance between the measurement sites^16^: aPWV (m/s)=0.8 (direct distance [m]/Δt). In the third center (Ankara, Turkey), the pulse‐wave velocity was measured with an arteriograph device (TensioMed Ltd, Software v. 1.9.9.2, Budapest, Hungary) and subsequently converted in SphygmoCor aPWV calculated using the direct distance scaled for 0.8 according to the relationship previously found by Ring et al^17^:

$${\text{PWV}}_{\mathrm{Arteriograph}}\;\left(\text{m/s}\right)=3.2846+0.6152\times {\text{aPWV}}_{\mathrm{SphygmoCor}}\;\left(\text{m/s}\right)$$

The annual progression in aPWV during follow‐up was calculated as: ΔaPWV (m/s per year)=(aPWV at the end of follow up−aPWV at baseline)/follow‐up duration (year).

### Disease Activity

Active disease was defined by Mayo Score ≥2 in subjects with UC and Harvey‐Bradshaw Index ≥5 in subjects with CD.^18^, ^19^

### Statistical Analysis

Data were analyzed with IBM SPSS Statistics version 19.0 (SPSS Inc, Chicago, IL). Continuous variables are presented as the means (SDs), and categorical variables are presented as percentages. Using ANOVA and the Bonferroni test for multiple comparisons, we determined a sample size adequate to demonstrate that aortic stiffening during follow‐up was lower in patients with IBD treated with anti‐TNFα than in control subjects. The study power (90%) was calculated to detect a significant (*P*<0.01) difference in aortic stiffening among groups, hypothesizing that ΔaPWV was −0.03±0.21 m/s per year in patients with IBD treated with anti‐TNFα, 0.20±0.15 m/s per year in those treated with salicylates, 0.02±0.21 m/s per year in those treated with other immunosuppressive drugs, and 0.08±0.09 m/s per year in control subjects, as previously reported in our pilot study.^14^ A sample size of 167 patients with IBD (29 patients treated with anti‐TNFα, 31 patients treated with salicylates, and 107 patients treated with other therapies) and 167 control subjects provided >90% power to detect a significant (*P*<0.01) difference in aortic stiffening between groups. These calculations were based on 10 000 Monte Carlo samples.

Clinical and hemodynamic variables were compared using ANOVA for continuous variables with the Bonferroni test for multiple comparisons and the chi‐square test for categorical variables in univariate analyses. Comparisons between measures at baseline and follow‐up were made using repeated‐measures ANOVA. Generalized estimating equations were used to examine correlations of annual progression (taken as the difference between the first and second visits divided by time between visits) in aPWV to conventional risk factors, inflammation, and therapy in patients with IBD. In generalized estimating equations, we used the center‐specific *z* score of ΔaPWV. Thus, effect estimates for each center reflect the change for a 1‐SD increase in ΔaPWV in patients with IBD. A center‐specific *z* score was calculated according to the following formula: *z* score=([individual value−population mean]/population SD), where the mean values and SD were calculated in each cohort. Covariates included were age, body mass index, mean BP, heart rate, total cholesterol, high‐density lipoprotein cholesterol, triglycerides, and active disease. Values of these variables at baseline and annual progression were included in the analysis as well as disease duration, immunosuppressive therapy, baseline aPWV, and center of origin. The contribution of these variables to ΔaPWV was first assessed in univariate and then in multivariate analysis adjusted for baseline measurements and the matching variables (age, sex, and mean BP). A 2‐tailed *P*<0.05 was considered significant.

The authors had full access to the data and take full responsibility for its integrity. All authors read and agreed to the manuscript as written.

## Results

### Clinical Characteristics of Patients With IBD

The main baseline clinical and demographic data of patients with IBD and matched control subjects are presented in Table 1. A total of 82 patients with UC, 85 patients with CD, and 167 matched control subjects were enrolled and followed for a median follow‐up of 4 years (range, 2.5–5.7 years). Mean follow‐up was comparable between patients with UC, patients with CD, and control subjects (*P*=0.71). The matching process worked well because age, sex, and mean BP were comparable between groups, as were systolic and diastolic BP. At baseline examination, patients with UC and those with CD had higher aPWV (7.8±2.0, 7.9±2.0, and 7.1±1.4 m/s, respectively; *P*<0.001) and heart rate (72±13, 72±13, and 68±11 beats/min, respectively; *P*<0.001) than did control subjects.

**Table 1:** Demographics and Baseline Characteristics of Patients With Inflammatory Bowel Disease and Age‐, Sex‐, and Mean Blood Pressure–Matched Control Subjects

Group	UC (n=82)	CD (n=85)	Controls (n=167)	*P* Value^a^	Groups Comparison^b^
	A	B	C		
Age, y	37 (11)	39 (13)	38 (12)	0.56	
Male sex, %	55	61	57	0.71	
Mean BP, mm Hg	87 (11)	89 (11)	87 (11)	0.39	
Systolic BP, mm Hg	117 (20)	120 (14)	118 (13)	0.43	
Diastolic BP, mm Hg	72 (10)	73 (12)	71 (11)	0.32	
Heart rate, beats/min	72 (13)	72 (13)	68 (11)	0.01	A≠C; B≠C
aPWV, m/s	7.8 (2.0)	7.9 (2.0)	7.1 (1.4)	<0.001	A≠C; B≠C
BMI, kg/m^2^	24 (5)	24 (4)	25 (5)	0.03	
hsCRP, log (mg/L)	0.96 (1.88)	1.03 (1.75)	0.48 (1.34)	0.02	B≠C
TC, mmol/L	4.08 (0.88)	4.47 (0.95)	4.56 (0.74)	<0.001	A≠B; A≠C
HDL, mmol/L	1.24 (0.40)	1.28 (0.40)	1.30 (0.26)	0.45	
TG, mmol/L	1.15 (0.70)	1.44 (0.69)	1.34 (0.85)	0.047	A≠B
Active disease, %	28	33	···	0.49	
Therapy				<0.001	
Salicylates, %	49	79	···		
Anti‐TNFα, %	27	8	···		
Other therapy, %	24	13	···		

Values represent mean (SD) or percentage. Anti‐TNFα indicates anti–tumor necrosis factor‐alpha; aPWV, aortic pulse‐wave velocity; BMI, body mass index; BP, blood pressure; CD, Crohn disease; hsCRP, high‐sensitivity C‐reactive protein; TC, total cholesterol; TG, triglycerides; UC, ulcerative colitis.

a As appropriate, chi‐square or ANOVA tests.

b Bonferroni test for multiple comparisons.

Current therapy for the IBD patients included anti‐TNFα therapy (alone or in association with other drugs, n=29), salicylates (alone or in association with other drugs except for anti‐TNFα therapy, n=107), and other disease‐modifying drugs (without anti‐TNFα therapy and salicylates, n=31). Most patients were taking 2 or more drugs concomitantly. Baseline and follow‐up characteristics of control subjects and patients with IBD sorted for the immunosuppressive therapy at baseline are reported in Table 2.

**Table 2:** Baseline and Follow‐Up Characteristics of Control Subjects and Patients With Inflammatory Bowel Disease Sorted for the Immunosuppressive Therapy at Baseline

	Control Subjects (n=167)		Patients With Inflammatory Bowel Disease						*P* Value^a^
			Salicylates (n=107)		Others (n=31)		Anti‐TNFα (n=29)		
	Baseline	Follow‐Up	Baseline	Follow‐Up	Baseline	Follow‐Up	Baseline	Follow‐Up	
Mean BP, mm Hg	87 (11)	87 (10)	89 (12)	88 (11)	86 (8)	83 (8)	87 (12)	86 (12)	0.11
Systolic BP, mm Hg	118 (13)	121 (13)	120 (16)	120 (17)	117 (11)	113 (11)	115 (25)	117 (16)	0.19
Diastolic BP, mm Hg	71 (11)	71 (10)	73 (12)	72 (10)	71 (9)	68 (9)	73 (10)	70 (11)	0.13
Heart rate, beats/min	68 (11)	69 (11)	72 (13)	72 (14)	70 (13)	71 (12)	75 (11)	71 (10)	0.05
aPWV, *z* score	7.1 (1.4)	7.4 (1.3)	7.8 (2.1)	8.0 (2.5)	7.5 (1.3)	7.5 (1.6)	8.5 (2.3)	7.9 (1.4)	0.02
BMI, kg/m^2^	25 (5)	25 (5)	23 (4)	24 (4)	25 (5)	25 (5)	23 (5)	24 (4)	0.03
hsCRP, log (mg/L)	0.48 (1.34)	0.43 (1.31)	0.67 (1.94)	0.77 (1.35)	1.69 (1.17)	1.53 (1.42)	1.34 (1.72)	1.23 (1.38)	0.76
TC, mmol/L	4.56 (0.74)	4.59 (0.74)	4.33 (0.96)	4.59 (1.05)	4.00 (0.72)	4.46 (1.01)	4.40 (0.99)	4.57 (0.85)	0.046

Values represent mean (SD). Anti‐TNFα indicates anti–tumor necrosis factor‐alpha; aPWV, aortic pulse‐wave velocity; BMI, body mass index; BP, blood pressure; hsCRP, high‐sensitivity C‐reactive protein; TC, total cholesterol.

a Visit‐therapy interaction.

### Inflammation and Aortic Stiffening in Patients With IBD

**Figure 1:**
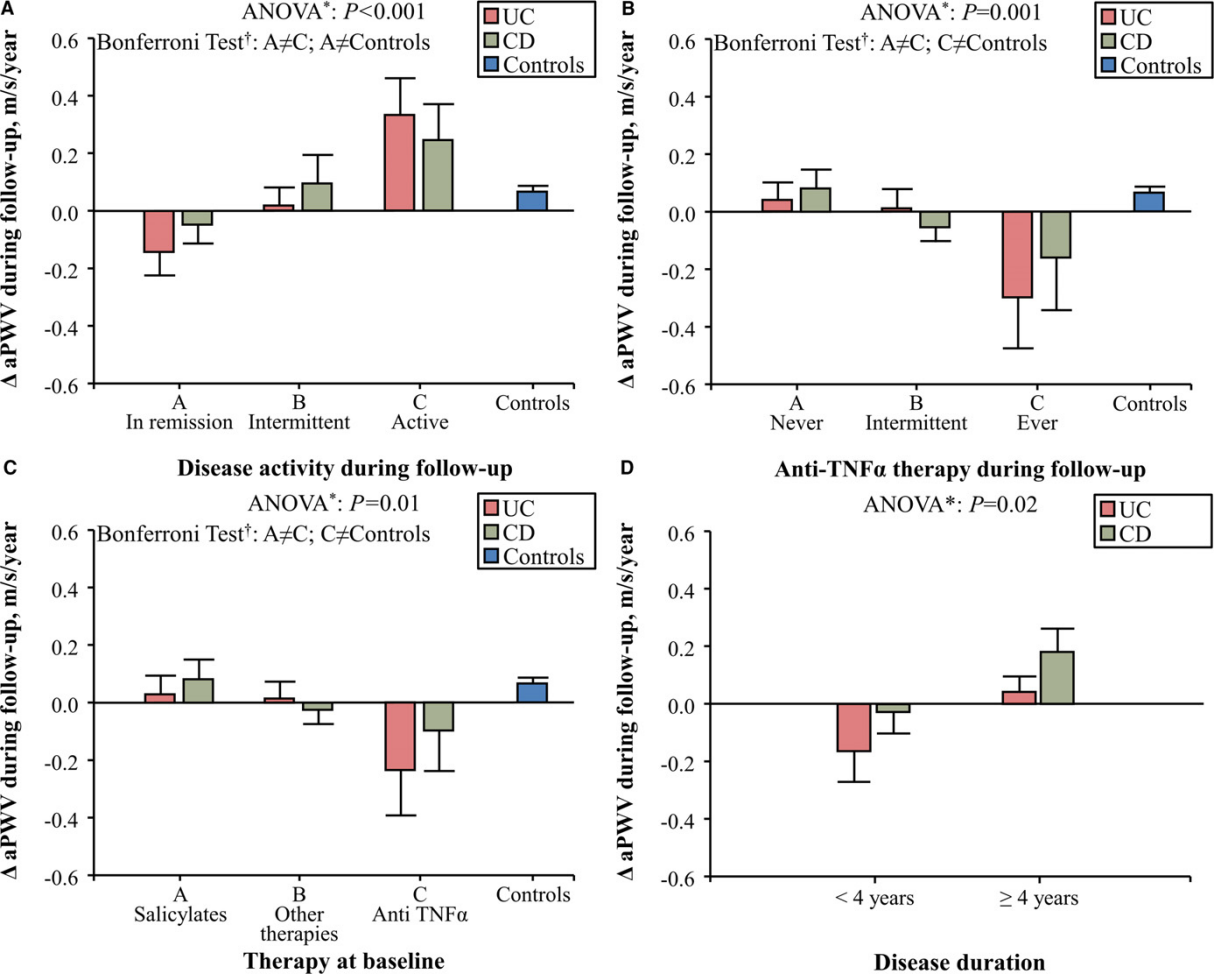
Changes in aortic pulse‐wave velocity (ΔaPWV) during follow‐up in patients with Crohn disease (CD) and in those with ulcerative colitis (UC) sorted for disease activity during follow‐up (**A**), anti–tumor necrosis factor‐alpha (anti‐TNFα) therapy during follow‐up (**B**), immunosuppressive therapy at baseline (**C**), and disease duration at baseline (**D**). Patients with inflammatory bowel disease (IBD) in remission during follow‐up and those treated with anti‐TNFα experienced an aortic destiffening during follow‐up, whereas aPWV increased in patients with active disease during follow‐up, in patients never treated with anti‐TNFα during follow‐up, and in those treated with salicylates. Bars represent means and SEMs. *Data of patients with CD and UC were analyzed as a whole group using repeated measures ANOVA. ^†^Bonferroni Test of within‐subject contrasts.

**Figure 2:**
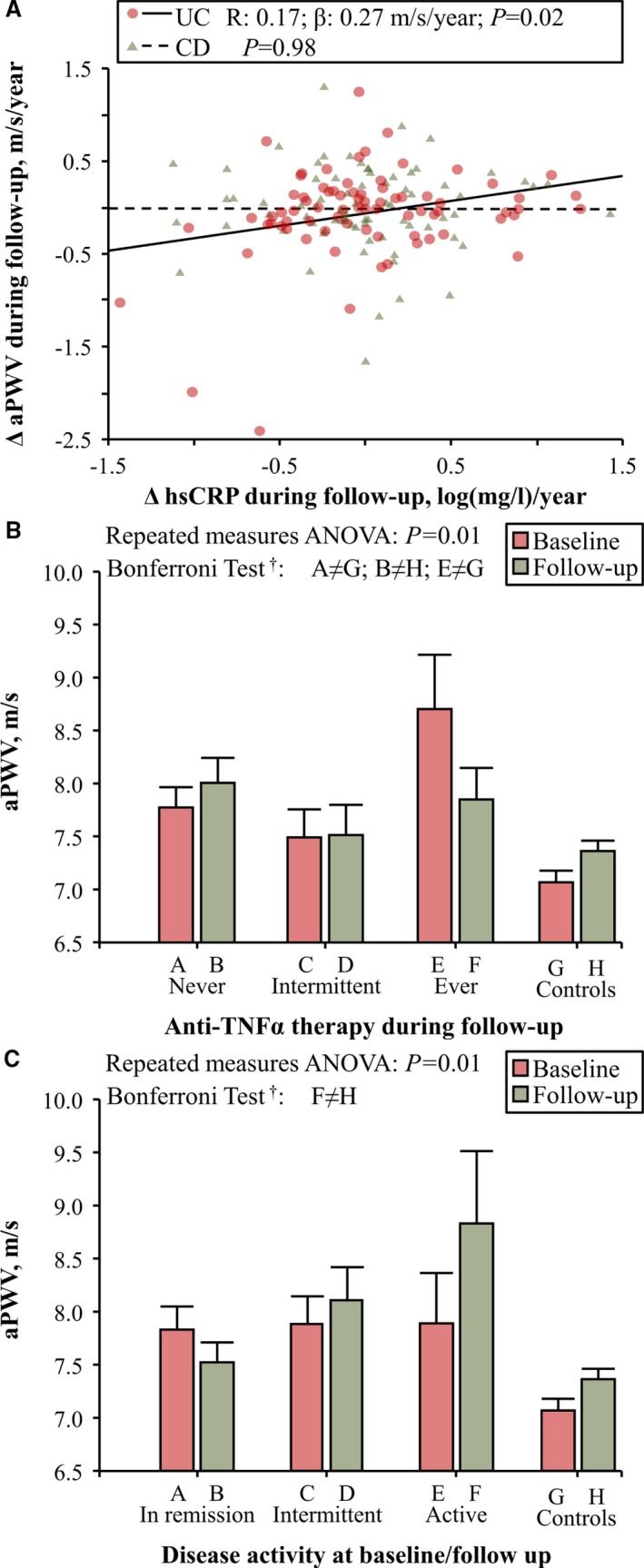
**A**, Changes in high‐sensitivity C‐reactive protein (ΔhsCRP) were correlated with aortic pulse‐wave velocity (ΔaPWV) during follow‐up in patients with ulcerative colitis (UC) but not in patients with Crohn disease (CD). **B**, Effect of therapy on aPWV in patients with inflammatory bowel disease (IBD; data of patients with CD and UC were analyzed as a whole group); aPWV was higher in patients never treated with anti‐TNFα during follow‐up than in control subjects at both baseline (Bonferroni test: A≠G) and follow‐up examination (Bonferroni test: B≠H), whereas in patients always treated with anti‐TNFα during follow‐up, aPWV was higher at baseline examination (Bonferroni test: E≠G) and reduced to a level comparable to that of control subjects at the end of follow‐up. **C**, Effect of disease activity on aPWV in patients with IBD (data of patients with CD and UC were analyzed as a whole group). At the end of follow‐up aPWV was higher in patients with active disease than in control subjects (Bonferroni test: F≠H). *Data were analyzed using repeated‐measures ANOVA). ^†^Bonferroni test of within‐subject contrasts.

Disease activity and immunosuppressive therapy influenced aortic stiffness, as patients with IBD in remission during follow‐up and those treated with anti‐TNFα experienced an aortic destiffening during follow‐up, whereas aPWV increased in patients with active disease during follow‐up, in patients never treated with anti‐TNFα during follow‐up, and in those treated with salicylates (Figure 1A through 1C). These results were confirmed in a multivariate generalized estimating equation model (Table 3). The duration of anti‐TNFα therapy was not associated with the magnitude of the reduction in aPWV at the end of follow‐up (*P*=0.85). Disease duration, a factor associated with the amount of chronic inflammation over time, was associated with aortic stiffening, whereas an increased baseline aPWV was associated with the largest reduction in aortic stiffness (Table 3). In patients with UC, an increase in high‐sensitivity C‐reactive protein during follow‐up was associated with aortic stiffening (Figure 2A). In a repeated‐measures ANOVA model, we reported that aPWV was reduced to a level comparable to that of control subjects after 4 years of treatment with anti‐TNFα therapy (Figure 2B) and that aPWV increased more in patients with active disease during follow‐up than in control subjects (Figure 2C).

**Table 3:** Univariate and Multivariate Generalized Estimating Equations: Relation of Annual Progression of aPWV to Traditional Cardiovascular Risk Factors, Inflammation, and Therapy in Patients With Inflammatory Bowel Disease

Independent Variable	Univariate Analysis		Multivariate Analysis	
	Beta (*z* Score)	*P* Value	Beta (*z* Score)	*P* Value
Baseline measures				
Age, y	0.002	0.79	0.15	0.03
Male sex, y/n	0.018	0.91	0.013	0.93
Mean BP, mm Hg	0.011	0.12	0.013	0.04
Heart rate, beats/min	−0.006	0.36		
Baseline aPWV, *z* score	−0.295	0.02	−0.403	<0.001
BMI, kg/m^2^	−0.001	0.97		
hsCRP, log (mg/L)	0.004	0.93		
TC, mmol/L	−0.025	0.74		
Active disease, y/n	0.383	0.02	0.744	<0.001
Disease duration, 5 years	0.267	0.003	0.241	<0.006
Crohn disease, y/n	0.255	0.10		
Immunosuppressive therapy at baseline				
Salicylates	0			
Others	−0.154	0.25	−0.290	0.02
Anti‐TNFα	−0.553	0.03	−0.376	0.04
Center of origin				
Catania	0			
Palermo	<0.001	>0.99		
Ankara	<0.001	>0.99		
Follow‐up measures				
ΔMean BP, mm Hg	0.058	0.18		
ΔHeart rate, beats/min	0.109	<0.001	0.087	<0.001
ΔBMI, kg/m^2^	−0.033	0.22		
ΔhsCRP, log (mg/L)	0.476	0.046		
ΔTC, mmol/L	−0.379	0.38		
ΔActive disease, y/n	1.866	<0.001	1.277	0.006
Anti‐TNFα therapy during follow‐up				
Never	0			
Intermittent	−0.134	0.34		
Ever	−0.684	0.02		

Center‐specific *z* score was calculated according to the following formula: *z* score=([individual value−population mean]/population SD) in patients with inflammatory bowel disease. Univariate analysis was performed using the enter method. The association with annual progression in dependent variables was adjusted for baseline values. Multivariable analysis included all variables considered in univariate analysis using backward method. ΔActive disease indicates annual progression in active disease; ΔAnti‐TNFα, annual progression in anti–tumor necrosis factor‐alpha therapy; ΔBMI, annual progression in body mass index; ΔHeart rate, annual progression in heart rate; ΔhsCRP, annual progression in high sensitivity C‐reactive protein; ΔMean BP, annual progression in mean blood pressure; ΔTC, annual progression in total cholesterol; anti‐TNFα, anti–tumor necrosis factor‐alpha therapy; aPWV, aortic pulse wave velocity; BMI, body mass index; BP, blood pressure; hsCRP, high‐sensitivity C‐reactive protein; TC, total cholesterol.

Finally, immunosuppressive therapy at baseline predicted the annual increase of aPWV during follow‐up (Figure 1C; Table 3).

## Discussion

To our knowledge, this is the first longitudinal study designed to evaluate with adequate power the long‐term effect of inflammation and immunosuppressive therapy on aPWV in patients with IBD.

Starting in 2012,^20^ a growing number of cross‐sectional studies, included in 2 aggregate data meta‐analyses performed by our group^9^, ^10^ and a third aggregate data meta‐analysis performed by an independent group,^11^ reported an increased aortic stiffness and reflection waves in patients with IBD. These findings were confirmed, as expected, in this study at baseline examination (Table 1). Moreover, we also reported that disease duration was associated with aortic stiffening even after adjustment for major confounders (Table 3). Considering that patients with longer disease duration were exposed to a significantly higher amount of inflammation over time than patients with short disease duration, our findings suggest that chronic inflammation leads to aortic stiffening in patients with IBD. Similar results were reported in patients with rheumatoid arthritis, another disease characterized by chronic severe inflammation.^21^

We also reported that patients with active disease were more prone to aortic stiffening than those in remission, in whom aortic stiffness is even reduced during follow‐up. These findings, paired with the association between the increase in high‐sensitivity C‐reactive protein during follow‐up and aortic stiffening in patients with UC and with the results of an individual participant data meta‐analysis of 4 cross‐sectional studies recently published by our group that observed an increased aPWV in patients with IBD and active disease,^12^ suggest that, at least in patients with UC, flares of acute inflammation can lead to aortic stiffening. Moreover, within patients with active disease at baseline and in remission at follow‐up, aPWV was reduced in patients with UC, whereas a mild and not significant increase of aPWV was reported in those with CD (Group B of Figure 1A); immunosuppressive therapy may have played a role in this process because, within these patients, only 18% of those with CD were treated with anti‐TNFα during follow‐up compared with 67% of patients with UC.

The next step of this longitudinal study was to test in patients with IBD whether aortic stiffening could be reversed by immunosuppressive therapy after a follow‐up of 4 years. Our data suggest that long‐term anti‐TNFα therapy reduces aortic stiffness in patients with IBD. This result agrees with our previous findings, one small pilot longitudinal study, and a meta‐regression analysis of cross‐sectional studies performed in patients with IBD,^9^, ^14^ and with similar evidence reported in another chronic inflammatory disorder.^15^ It remains to be clarified whether anti‐TNFα therapy is associated with an improvement of functional (ie, endothelial dysfunction) and/or structural arterial stiffening (alterations of the arterial wall structure, intima‐media thickening).^22^ We have recently hypothesized that anti‐TNFα therapy could have a beneficial effect on functional arterial stiffening in patients with rheumatoid arthritis.^23^ The lack of association between the duration of anti‐TNFα therapy and the magnitude of the reduction in aPWV observed in this longitudinal study in patients with IBD and the largest aortic destiffening during follow‐up observed in patients with a recent diagnosis of IBD (<4 years) (Figure 1D) could suggest that treatment with anti‐TNFα has a beneficial effect on functional arterial stiffening. Further studies are needed to confirm this hypothesis.

Finally, we reported that aPWV increased in IBD patients treated with salicylates. This result agrees with our pilot longitudinal study^14^ and with the results of a meta‐regression analysis of cross‐sectional studies,^24^ in which we hypothesized a lack of efficacy of salicylates in protecting IBD patients from arterial stiffening. We can speculate that the long‐term use of a high dose of salicylates could have led to vasoconstriction in patients with IBD.^25^ Alternatively, together with the limited efficacy in the maintenance of remission in patients with CD already reported in current guidelines,^26^ we can also hypothesize that a therapy based on salicylates is ineffective to reduce inflammation in patients with IBD and raise questions about their role as maintenance therapy.

### Methodological Issues

This study has some potential limitations. First, this is an observational study, and therefore the assignment of immunosuppressive treatment to patients with IBD is, by definition, not randomized. This reflects the fact that it was considered unethical to conduct a double‐blind, randomized trial of anti‐TNFα therapy in IBD patients who have not responded to other therapies. A similar study design was adopted previously by our group and others.^14^, ^27^, ^28^, ^29^ As already done in our previously published pilot study,^14^ we attempted to minimize bias by using (1) a physician unfamiliar with the study for the prescription of the therapy, (2) a blinded operator for the measurement of hemodynamic parameters, (3) a second blinded operator for the collection of the clinical data using a standardized questionnaire, and (4) a matching strategy. However, due to the nonrandomized design of the present study, we cannot exclude the possibility of a non–drug‐related reduction in aPWV. Second, the intima‐media thickness and endothelial function were not evaluated in this study. Therefore, we can only speculate on the effect of inflammation and immunosuppressive therapy on functional and structural arterial stiffening. Third, thanks to the multicenter design, we enrolled and followed for 4 years 167 patients with IBD and 167 matched control subjects. However, despite the fact that the sample size was adequate to demonstrate that aortic stiffening during follow‐up was lower in patients with IBD treated with anti‐TNFα than in control subjects, this study was not designed to perform subgroup analyses in patients with UC and CD. This was mostly because it was difficult for us to gather and follow more patients for 4 years. Finally, 2 devices were used to measure the pulse‐wave velocity, a SphygmoCor device (SphygmoCor system; AtCor Medical, Sydney, Australia) in 2 centers (Catania and Palermo, Italy) and an arteriograph device (TensioMed Ltd, Budapest, Hungary) in the third center (Ankara, Turkey). We attempted to reduce the technique‐related effect converting the arteriograph pulse‐wave velocity in SphygmoCor aPWV calculated using the direct distance scaled for 0.8 according to the relationship previously found by Ring et al^17^; this procedure was successful because ΔaPWV was not related to the center of origin (*P*=0.36) in 1‐way ANOVA. Moreover, the use of center‐specific *z* score of ΔaPWV in generalized estimating equations allowed us to reduce further the source of variability of ΔaPWV related to the center of origin (*P*>0.99; Table 3).

## Conclusions

Aortic stiffening is evident in patients with IBD as a consequence of chronic inflammation. During a follow‐up of 4 years, patients treated with anti‐TNFα experienced a reduction of aPWV, an established surrogate measure of risk, to a level comparable to that of healthy individuals. This suggests that effective long‐term control of inflammation may reduce cardiovascular risk in patients with IBD.

## Author Contributions

All authors meet the International Committee of Medical Journal Editors’ recommendations for authorship credit. Drs Zanoli and Boutouyrie contributed substantially to the conception and design of the study, analysis, and interpretation of data and drafting the paper; Drs Mulé, Cottone, Perna, Fatuzzo, Castellino, and Laurent contributed substantially to the design of the study, interpretation of data, and revising the work critically for important intellectual content; Drs Ozturk, Cappello, Inserra, Tuttolomondo, Torres, Pinto, Aykan, Geraci, Duminuco, and Riguccio contributed substantially to the acquisition of data and revising the work critically for important intellectual content. All authors read and approved the manuscript and agreed to be accountable for all aspects of the work in ensuring that questions related to the accuracy or integrity of any part of the work are appropriately investigated and resolved.

## Sources of Funding

This study was partially funded by the Ministry of Health, Italy (GR‐2011‐02349066) and by 2016/2018 Department Research Plan of University of Catania, Department of Clinical and Experimental Medicine (Project #A).

## Disclosures

None.
